# Transcriptomics reveal the genetic coordination of early defense to *Armillaria* root rot (ARR) in *Prunus* spp

**DOI:** 10.3389/fpls.2023.1181153

**Published:** 2023-06-02

**Authors:** Lichun Cai, Jeffrey Adelberg, Jacqueline Naylor-Adelberg, Guido Schnabel, Alejandro Calle, Zhigang Li, Gregory Reighard, Ksenija Gasic, Christopher A. Saski

**Affiliations:** Department of Plant and Environmental Sciences, Clemson University, Clemson, SC, United States

**Keywords:** *Armillaria* root rot, transcriptomics, *Prunus*, rootstock, fungi

## Abstract

*Armillaria* root rot (ARR) poses a significant threat to the long-term productivity of stone-fruit and nut crops in the predominant production area of the United States. To mitigate this issue, the development of ARR-resistant and horticulturally-acceptable rootstocks is a crucial step towards the maintenance of production sustainability. To date, genetic resistance to ARR has been found in exotic plum germplasm and a peach/plum hybrid rootstock, ’MP-29‘. However, the widely-used peach rootstock Guardian® is susceptible to the pathogen. To understand the molecular defense mechanisms involved in ARR resistance in *Prunus* rootstocks, transcriptomic analyses of one susceptible and two resistant *Prunus* spp. were performed using two causal agents of ARR, including *Armillaria mellea* and *Desarmillaria tabescens*. The results of *in vitro* co-culture experiments revealed that the two resistant genotypes showed different temporal response dynamics and fungus-specific responses, as seen in the genetic response. Gene expression analysis over time indicated an enrichment of defense-related ontologies, including glucosyltransferase activity, monooxygenase activity, glutathione transferase activity, and peroxidase activity. Differential gene expression and co-expression network analysis highlighted key hub genes involved in the sensing and enzymatic degradation of chitin, GSTs, oxidoreductases, transcription factors, and biochemical pathways likely involved in *Armillaria* resistance. These data provide valuable resources for the improvement of ARR resistance in *Prunus* rootstocks through breeding.

## 1 Introduction

Plants have evolved an intricate and unique immune system to resist colonization from pathogens such as viruses, fungi, and bacteria (Jones and Dangl, 2006). Unlike mammalian cells, plants lack circulatory immune cells and a somatic adaptive immune system to detect invaders (Jones and Dangl, 2006). Plant immune systems are seemingly far less complex; however, plant immune responses are precise and often create a persistent memory of the encountered pathogen, resembling vertebrate immunity features (Spoel and Dong, 2012). Plants rely on two layers of their innate immune system to recognize and respond to pathogen invasions which include pattern-triggered immunity (PTI) and effector-triggered immunity (ETI) (Jones and Dangl, 2006; Thomma et al., 2011; Spoel and Dong, 2012; Cui et al., 2015; Peng et al., 2018). The plant cell wall surface is the first line of plant defense and contains pattern recognition receptors (PRRs) that can detect microorganism-associated molecular patterns (MAMPS) – such as lipopolysaccharides, peptidoglycans, and bacterial flagellin (Spoel and Dong, 2012) to activate a defense response against the invading pathogens (Jones and Dangl, 2006; Zipfel, 2014). Typical plant PRRs are described as families of genes belonging to Receptor-like kinases (RLKs, also knowns as receptor kinases) and receptor-like proteins (RLPs) (Boller and Felix, 2009). Typical RLKs have an extracellular domain for ligand detection, transmembrane, and intracellular kinase domains (Jones and Dangl, 2006; Zipfel, 2014; Zipfel and Oldroyd, 2017). Furthermore, RLPs are essentially RLKs that lack the kinase domain (Jones and Dangl, 2006; Zipfel, 2014; Couto and Zipfel, 2016; Zipfel and Oldroyd, 2017). Plant receptors comprised of these motifs have evolved extracellular domains that recognize a wide range of bacterial ligands (Yu et al., 2017). The other critical component of plant immunity, ETI, is a result of co-evolution with pathogens (Spoel and Dong, 2012). This type of immunity is induced by pathogen-produced effector molecules that trigger host-encoded resistance proteins (R genes). Resistance genes sense changes in host signaling networks (or through direct binding) that can initiate a hypersensitive response leading to programmed cell death of the infected cells and the production of phytoanticipins limiting pathogen spread and local resistance (Spoel and Dong, 2012; Kourelis and van der Hoorn, 2018). Characteristic signatures of R genes include nucleotide binding-site leucine-rich repeats (NBS-LRR) (Jones and Dangl, 2006; Han, 2019), coiled-coil domains (Dangl and Jones, 2001; Jones and Dangl, 2006), TIR (toll/interleukin-1 receptors) and LRR-like domains, and kinase domains (Dangl and Jones, 2001; Jones and Dangl, 2006). Many R-genes are found in clusters on the genome, which may reflect their common ancestry and/or functional redundancy (Chen et al., 2020; Mizuno et al., 2020).

*Armillaria* root rot (ARR) is a severe threat to many economically significant stone fruit and nut crops throughout the U.S. The pathogenic fungi responsible for ARR consist of three geographically isolated species: *Armillaria mellea* (Vahl) P. Kumm in California, *A. solidipes* Peck (=*A. ostoyae* (Romag.) Herink) in Michigan, and *Desarmillaria tabescens* (Scop.) R. A. Koch & Aime comb. nov. in the southeast (Proffer et al., 1988; Schnabel et al., 2005). As facultative necrotrophs, these fungi initially enter the host root cambium through close root-to-root contact *via* fungal mycelium and/or through rhizomorph extensions of the hyphae (Baumgartner et al., 2011; Cleary et al., 2012) and then live as a saprophyte consuming the dead root tissue as its source of nutrition (Baumgartner et al., 2011). During this phase, a characteristic white rot is typically observed as *Armillaria* spp. decomposes the host plant cell wall components (Coetzee et al., 2011; Devkota and Hammerschmidt, 2020). This infection process often kills the host during its most productive phase, which greatly reduces the lifespan of what should be long-lived perennial crops. Furthermore, *Armillaria* spp. can survive in roots in the soil in a vegetative state for years to decades and serve as inoculum for future replantings (Baumgartner and Rizzo, 2002). *Armillaria* spp. also has a broad host range that includes many other tree fruit species plus important forest tree species, most notably oak trees (Raabe, 1962; Hood, 1991). Many factors contribute to the ARR epidemic and the subsequent rapid spread of the disease in production sites. *Armillaria* can spread effectively from tree to tree with an average spread per year of 0.2 (Vanderkamp, 1993) to 1 m (Peet et al., 1996) and can also travel from orchard to orchard on tilling equipment. These characteristics make this pathogen challenging to manage and control.

ARR disease management has been ongoing for well over fifty years with limited success. The most promising cultural practice, termed root collar excavation (Schnabel et al., 2012), includes planting the trees shallow on a soil berm to allow the roots to establish for several years before removing the berm leaving the tree taproot aboveground. The inability of the fungus to grow above the soil line prevents lower crown colonization by the infected root (Schnabel et al., 2012). This practice extends orchard life by about two years but is not a long-term solution, such as genetic resistance, leaving the development of tolerant rootstocks a priority. Among the *Prunus* crops, plum species have exhibited the highest natural resistance to ARR (Guillaumin et al., 1991). In pursuit of this resistance, interspecific crosses have been developed over the years to confer this resistance to susceptible *Prunus* species while achieving graft compatibility and other necessary production traits. Two interspecific resistant rootstocks, ‘Sharpe’ (Beckman et al., 2008) and ‘MP-29’ (Beckman et al., 2012), that are graft compatible with peach scions have been developed. However, both rootstocks have major drawbacks. For example, ‘Sharpe’ is susceptible to peach tree short life syndrome (PTSL) and reduces tree vigor and fruit size compared to peach seedling-type rootstocks (Beckman et al., 2008). The interspecific plum-peach rootstock ‘MP-29’ is resistant to both ARR and PTSL but, is a devigorating rootstock for peach scion productivity (Beckman et al., 2012). Furthermore, ‘MP-29’ is challenging to propagate in the nursery, so grower demands for this rootstock routinely go unmet. Disease screening in the field can take decades and is challenging because of the uneven distribution of inoculum loads in an ARR ‘hot’ site (Raabe, 1979). Greenhouse challenge assays have been developed, but reproducibility has been difficult (Mansilla et al., 2001; Raziq and Fox, 2005). An *in vitro*, agar-based method that greatly accelerated the screening process and significantly removed error margins from the process was more recently presented (Baumgartner et al., 2018). This screening system can determine resistance/susceptibly phenotypes within several months. Still, a primary drawback is that the roots were maintained in an anoxic environment leaving the roots non-lignified (herbaceous), and lacking some structures of well-developed root tissues, which may, in turn, influence the phenotypic outcome (Adelberg et al., 2021).

The present study investigates the transcriptional responses of three genotypes ‘MP-29’ (ARR resistant hybrid rootstock), *Prunus cerasifera* (plum species with known ARR genetic resistance), and ‘Guardian®’ (ARR susceptible peach rootstock) when challenged with *A. mellea* and *D. tabescens* in an advanced *in vitro* system (Adelberg et al., 2021). This system uses an aerated substrate, Oasis^®^ IVE, for improved root development that mimics field-produced roots and a larger vessel that allows for simultaneous growth of multiple plants (Adelberg et al., 2021; Parris et al., 2022). This research aims to identify biochemical pathways, gene regulatory networks, and the genetic coordination of resistance to ARR in various *Prunus* genotypes.

## 2 Materials and methods

### 2.1 Plant materials and fungal inoculation

Three *Prunus* genotypes, *P. persica* (Guardian®), *P. cerasifera* (14-4) and the peach plum hybrid (MP-29) with contrasting performance on resistance to ARR were used in this study. ‘Guardian®’ is susceptible to ARR while ‘14-4’ and ‘MP-29’ exhibited different levels of resistance to ARR. Plant material was inoculated with *A. mellea* and *D. tabescens* following protocol described in Adelberg et al. (2021). In short, agar-based stock plants, established from the aseptic cultures, were rooted in phenolic foam Oasis^®^ IVE (Smithers-Oasis Company, Kent, OH) housed in the RV 750 rectangular polycarbonate culture vessels (EightomegFIVE, Santa Paula, CA). The fungal inoculum for each species *A. mellea* and *D. tabescens* was prepared in the IVE foam following protocol described in Adelberg et al. (2021).

### 2.2 ARR disease scoring and sample collection

Plant response to ARR inoculation was scored on a weekly basis after co-culture assembly, using previously defined scoring scale from 0-5 (5, no symptoms; 4, a few leaves with necrotic tips; 3, half of the leaves showing necrosis; 2, half of the leaves were dead and widespread necrosis on the others; 1, almost the entire plant was dead; and 0, the whole plant was dead) (Adelberg et al., 2021). Root tissues sampled at 72 hours, 2 weeks, 5 weeks, and 8 weeks post inoculation were collected for RNA extraction. Each timepoint had three biological replicates drawn from the 15 plants in each vessel. Timepoints had been assigned to four vessels for each of the three genotypes, in the three fungus treatments (*A. mellea*, *D. tabescens*, Control). At each harvest stage, roots were carefully cut out of the IVE foam matrix with a scalpel, gently blotted dry and input into a cryovial, and immediately flash frozen in liquid nitrogen and stored at -80°C for RNA isolation.

### 2.3 RNA isolation, library construction and Illumina sequencing

Total RNA was extracted from each sample following the methods of Meisel et al. (2005). Total RNA was analyzed for quality and integrity *via* UV spectroscopy (Nanodrop8000 ThermoFisher Scientific) and Bioanalyzer 2100 (Agilent), respectively. All samples had a minimum RNA integrity score of 7. Total RNA was quantified using a double-stranded dye binding assay on the qubit (ThermoFisher Scientific). Library preparation was conducted with the NEBNext Ultra II RNA Library Prep Kit for Illumina following the manufacturer’s recommended procedures and pooled in equimolar ratios for sequencing. Paired-end reads for each sample (2×150bp) were collected on an Illumina NovaSeq 6000 S4 flow cell to an approximate depth of 40 million read pairs. Raw sequence reads were preprocessed for low-quality bases and adapter sequences with the Trimmomatic software (Bolger et al., 2014). Clean reads were then mapped to the *Armillaria* reference genome (Sipos et al., 2018) to identify and remove reads with fungal origin.

### 2.4 *De novo* transcriptome assembly and annotation

For each rootstock, a *de novo* transcriptome was assembled from the clean reads using the Trinity (v2.9.1) *de novo* assembler (Grabherr et al., 2011). The *de novo* transcriptomes for each genotype were assessed for completeness using the BUSCO (Simao et al., 2015) software and the embryophyte_odb9 (1,440 conserved genes) single-copy ortholog dataset. Transcripts were stringently assessed for genuine coding sequence with the TransDecoder software (Haas, 2021a) and only transcripts with protein sequences predicted were kept for further analysis. Trinotate pipeline (v3.2.0) was used to functionally annotate the final transcriptome for each genotype (Haas, 2021b). The databases used in the Trinotate pipeline include NCBI, SwissProt, HMMER, PFAM, singalP, tmHMM, GO and eggNOG. Principal components and co-expression analyses were only performed in ‘MP-29,’ for which data were available for all time points and treatments.

### 2.5 Differential gene expression analysis

Differential expression analysis was conducted using the Trinity RNA-seq analysis pipeline (Haas et al., 2013). In brief, preprocessed reads for each genotype were mapped to each respective reference transcriptome with the ‘Bowtie2’ short read aligner. Expectation-Maximization (RSEM) method was used for transcript abundance estimation. The raw count data were normalized with trimmed mean of means (TMM) method. Principal component analysis was performed to determine the relatedness of biological replicates. Differentially expressed genes (DEGs) between control group and treatment group were determined with edgeR package (Robinson et al., 2010). The final list of differentially expressed genes were filtered for a false discovery rate < 0.01 and an absolute value of the log2 (fold change) > 2.

### 2.6 Gene ontology enrichment analysis

Gene ontology enrichment analysis was performed using the clusterProfiler R package (Wu et al., 2021). GO annotation of each gene was extracted from the output of Trinotate pipeline, and related terms with similar annotations were merged to reduce redundancy. GO terms that were significantly enriched for DEGs were identified by comparing with the whole genome background based on a false discovery rate (FDR) < 0.01. P-values were adjusted for multiple comparisons using the Benjamini & Hochberg (BH) method (Ferreira, 2007).

### 2.7 Weighted gene co-expression network analysis

The co-expression network analysis was performed using Weighted Gene Co-expression Network Analysis (WGCNA) (Langfelder and Horvath, 2008). This analysis identifies genes with highly similar transcriptional patters and classifies them into co-expression modules. The normalized read counts were used to calculate adjacency matrices, and a soft thresholding power of six was used. The adjacency matrices were used to calculate topological overlap dissimilarity matrices which were subsequently used for estimating gene clustering trees. The minimum number of genes for the cluster is set to 50. After a module was identified, gene expression information within the module was used to estimate the module eigengene. The key modules associated with different timepoint/treatment combinations were identified based on module eigengene, which was the first principal component of a given module. The identified modules were used to identify hub-genes associated with biological processes of interest. From each module, the top five genes were considered as the hub genes based on the values of connectivity and gene significance obtained from WGCNA. The interaction network of hub-genes in each module was visualized using Cytoscape v3.9.1 (Shannon et al., 2003). Differential expression analysis and weighted gene co-expression network analysis were used to identify modules associated with host immunity and defense-related genes.

### 2.8 Identification of R-genes and orthologs between different *Prunus* species

The identification of orthologs between different species was performed by BLAST approach based on the reciprocal best hits (RBHs) by default parameters. Two transcriptomes were compared in a pairwise manner using amino acid sequences. To find orthologs as RBHs we sorted the BLAST hits from highest to lowest bit-scores, if the bit-scores were identical, and from smallest to highest E-values. The first hit within the sorted data was identified as the best hit. We also performed ortholog analysis with OrthoFinder software (https://github.com/davidemms/OrthoFinder) to identify the orthogroups among three *Prunus* species by default parameters. We detected nucleotide-binding site, leucine-rich repeat (NBS–LRR) domains by searching the interproscan output for the protein family ID PF00931.

## 3 Results

### 3.1 Defense responses to fungal infection (*A. mellea* and *D. tabescens*) in *Prunus* spp

**Figure 1:**
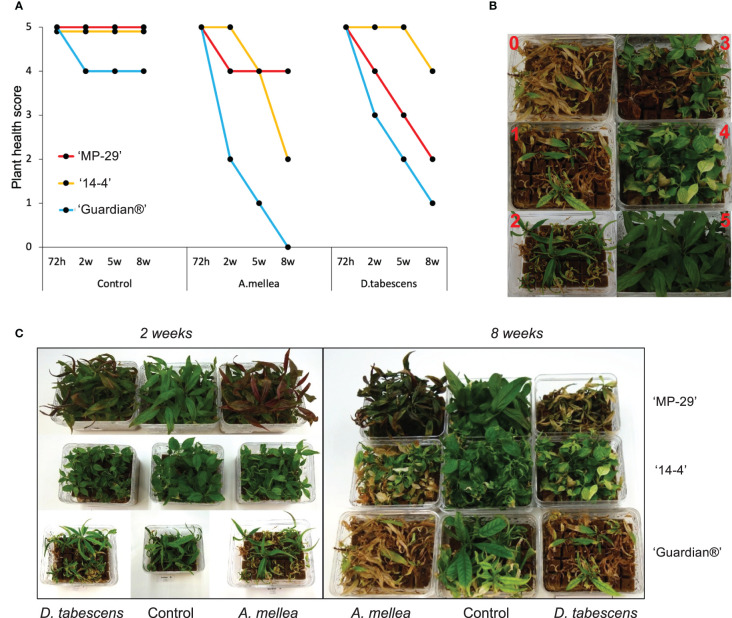
Defense responses to fungal infection in Prunus spp. **(A)** Variation in ARR resistance of three Prunus genotypes (‘MP-29’, ‘14-4’ and ‘Guardian®’) at different time points (72 hours, 2 weeks, 5 weeks, and 8 weeks) after being inoculated with A. mellea and D. tabescens. **(B)** The plants were evaluated using a disease rating scale, where 0 represents the complete death of the plant and 5 represents the absence of symptoms. The scale also includes intermediate stages of disease severity, such as a few leaves with necrotic tips (4), half of the leaves showing necrosis (3), half of the leaves dead and widespread necrosis on the others (2), and almost the entire plant dead (1). **(C)** Plant performance after fungal infection in three genotypes, illustrated at week 2 and 8.

Defense responses to fungal infection manifest differentially over time in the three *Prunus* genotypes (**Figure 1A**). All genotypes remained asymptomatic at ‘72h’ for both *A. mellea* and *D. tabescens* infections. ‘Guardian®’ (known ARR susceptible rootstock) began to show a decline in plant health at the ‘2w’ timepoint, with chlorosis and necrotic leaf tips as foliar symptoms which continued to progress during the time course when compared to ‘MP-29’ and ‘14-4’. For the *A. mellea* infection, ‘MP-29’ displayed resistance for eight weeks, while ‘14-4’ declined in health over the latter weeks but did display a resistance profile when compared to ‘Guardian®’. Conversely, ‘14-4’ exhibited substantial resistance for *D. tabescens* infection while ‘MP-29’ showed a continuous decrease in plant health (**Figure 1B, C**). The results of the disease rating analysis revealed that ‘Guardian®’ was susceptible to both fungi. Furthermore, ‘MP-29’ exhibited a higher level of resistance to *A. mellea* compared to ‘14-4’, which showed a higher level of resistance to *D. tabescens*, indicating that the genotypes have fungus-specific responses. Overall, insight into the temporal dynamics of the resistance to these fungi suggest that both ‘MP-29’ and ‘14-4’ are more resistant than ‘Guardian®’ (**Figure 1**).

### 3.2 High-quality *de novo* transcriptome assembly for ‘MP-29’, ‘14-4’ (*P. cerasifera*) and ‘Guardian®’

A *de novo* transcriptomics approach was used to profile gene expression in a genotype-specific manner for the three *Prunus* genotypes. Approximately 368 Gb raw data were generated for each genotype, ‘Guardian®’, ‘MP-29’ and ‘14-4’, respectively. Clean reads comprised ~94% of the data, ranging from 11.3 to 17.4 Gb per library (**Supplementary Table 1**). As expected, most fungal infected samples contained a high proportion of sequences derived from fungal RNA, including ‘Guardian®’ at 2-weeks treated by *A. mellea*, ‘Guardian®’ at 5-weeks treated by *D. tabescens*, ‘MP-29’ at 2-weeks treated by *A. mellea*, ‘MP-29’ at 2-weeks treated by *D. tabescens* and ‘14-4’ at 2-weeks treated by *A. mellea*. Fungal reads were binned, and the final dataset used for transcriptome assembling were 286, 426 and 240 Gb for ‘Guardian®’, ‘MP-29’ and ‘14-4’, respectively (**Supplementary Table 1**). Following the general workflow of the Trinity pipeline, a *de novo* transcriptome was assembled independently for each of three *Prunus* genotypes. Clean reads were assembled as contigs and further refined into unigenes. A total of 146,710, 148,398, and 110,743 transcripts were generated for ‘MP-29’, ‘14-4’ and ‘Guardian®’, respectively. These transcripts correspond to 43,936, 49,706 and 29,096 primary unigenes (**Table 1**). The unigene content for ‘MP-29’ and ‘14-4’ was almost doubled to that of ‘Guardian®’, corroborating that ‘MP-29’ is an interspecific hybrid, and suggesting that ‘14-4’ may also be as well. Single-copy gene ortholog content was assessed with BUSCO using the embryophyta_odb10 database and the results indicated a high level of completeness for all three genotypes with 93.7%, 93.5% and 95.8% for ‘MP-29’, ‘14-4’ and ‘Guardian®’, respectively (**Table 1**). Functional annotations were assigned to most of the predicted proteins.

**Table 1:** Statistics of assembled transcriptomes for ‘MP-29’, ‘14-4’ and ‘Guardian®’.

Genotype/statistics	MP29	14-4	Guardian®
Number of transcripts	146,710	148,398	110,743
Average transcript length (bp)	2,094	2,332	2,618
N50 transcript length (bp)	2,891	3,253	3,386
Number of unigenes	43,936	49,706	29,096
Average unigene length (bp)	1,581	1,637	2,025
N50 unigene length (bp)	2,764	2,754	3,300
GC content (%)	42.03	42.29	41.51
BUSCO value (%)	93.7	93.5	95.8

### 3.3 Differentially expressed genes in ‘MP-29’ (control vs. inoculation)

**Figure 2:**
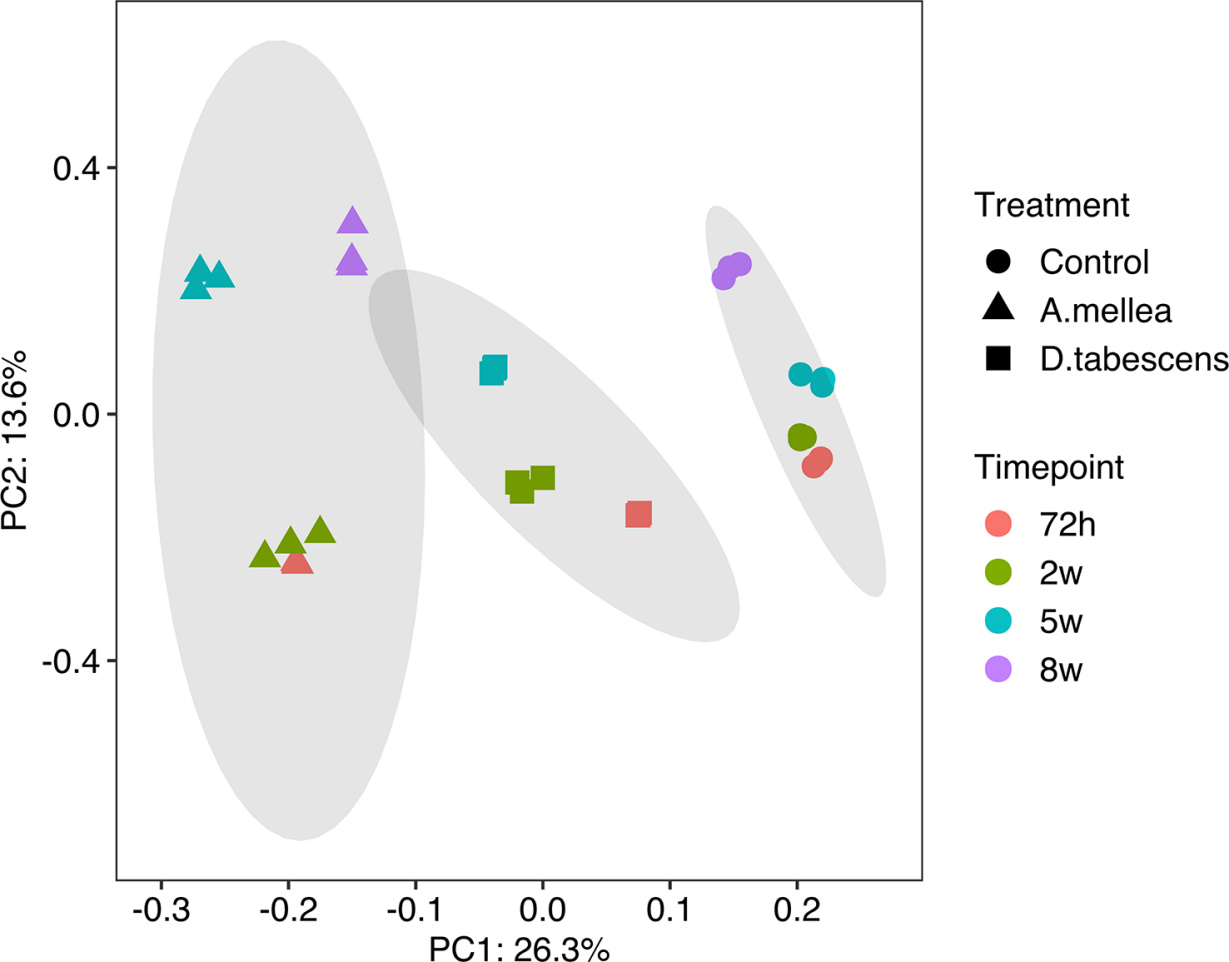
Principal component analysis (PCA) of gene expression in ‘MP-29’.

To investigate the molecular mechanisms underlying the defense response to *Armillaria/Desarmillaria* fungi in *Prunus* genotypes, a comprehensive analysis of gene expression was performed in ‘MP-29’. This was done because the available datasets for ‘MP-29’ were the most complete with all time points and treatments available (except *D. tabescens* treatment at 8 weeks). A Principal component analysis (PCA) of the transcriptome data from infected and uninfected samples over time showed that the first principal component largely reflected the fungal treatments, while the second principal component largely reflected the time post-inoculation, indicating that the treatment response was the more significant variable (**Figure 2**). The uninoculated (control) and two-fungal treated samples were clearly separated according to PC1. For each treatment, samples at 72 hours and 2 weeks appeared to be clustered together according to PC2. In contrast, subsequent time points exhibited differential transcriptional responses to infection, as indicated by PC2. The biological replicates for each time point and condition clustered exceptionally well, underscoring the value of the experimental control offered by the *in vitro* system (Adelberg et al., 2021).

**Figure 3:**
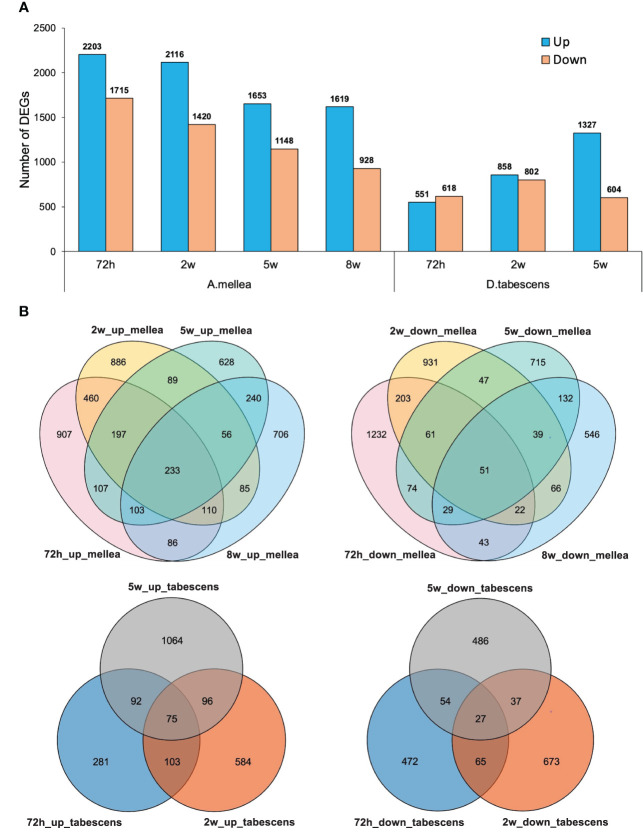
Differential transcriptomic responses to fungal infections. **(A)** Number of differentially expressed genes (P value < 0.01, log2(fold change) > 2) in each treatment/time point for ‘MP-29’. **(B)** Venn diagrams comparing DEGs between timepoints within treatment.

To further investigate the differentially expressed genes (DEGs) associated with ARR in ‘MP-29’, pairwise gene expression profiles of treated and untreated samples were analyzed at each time point. The results of the differential expression analysis indicate that a higher number of DEGs were identified for *A. mellea* inoculated samples compared to *D. tabescens* inoculated samples (**Figure 3**, **Supplementary File 1**). Specifically, a total of 3,918 transcripts were differentially expressed (2,203 up-regulated and 1,715 down-regulated) at 72 hours post-inoculation with *A. mellea*, indicating a rapid and strong response to infection in ‘MP-29’. The number of DEGs decreased over time for *A. mellea* treatment, with 2,547 DEGs identified at the final time point (**Figure 3A**). Additionally, 233 and 51 DEGs were consistently up-regulated and down-regulated across all time points, respectively. For *D. tabescens* treatment, the number of DEGs increased over time, with the most (1,931) identified at the final time point. Furthermore, 75 and 27 DEGs were consistently up- and down-regulated, respectively (**Figure 3B**).

To investigate the variations in the response of ‘MP-29’ to *A. mellea* and *D. tabescens*, we compared the DEGs identified in each treatment across all time points. A higher number of DEGs were identified in the *A. mellea* treatment than the *D. tabescens* treatment. Specifically, a total of 1,876, 1,612, and 1,047 unique DEGs were identified for *A. mellea* treatment at 72 hours, 2 weeks, and 5 weeks, respectively. Additionally, 325, 503, and 604 DEGs were commonly up-regulated between the two fungal treatments at 72 hours, 2 weeks, and 5 weeks, respectively (**Supplementary Figure 1**). Furthermore, we observed that 46 DEGs were consistently up-regulated across different treatments and time points (**Supplementary Table 2**, **Supplementary File 1**). These genes were annotated as encoding GSTs, hydrolases, and ribonucleases, which may play a role in ARR resistance. Overall, these results indicate that ‘MP-29’ exhibits different responses to *A. mellea* and *D. tabescens* based on the high number of unique DEGs identified between two treatments, which is consistent with the disease rating results.

### 3.4 Functional enrichment analysis of DEGs

**Figure 4:**
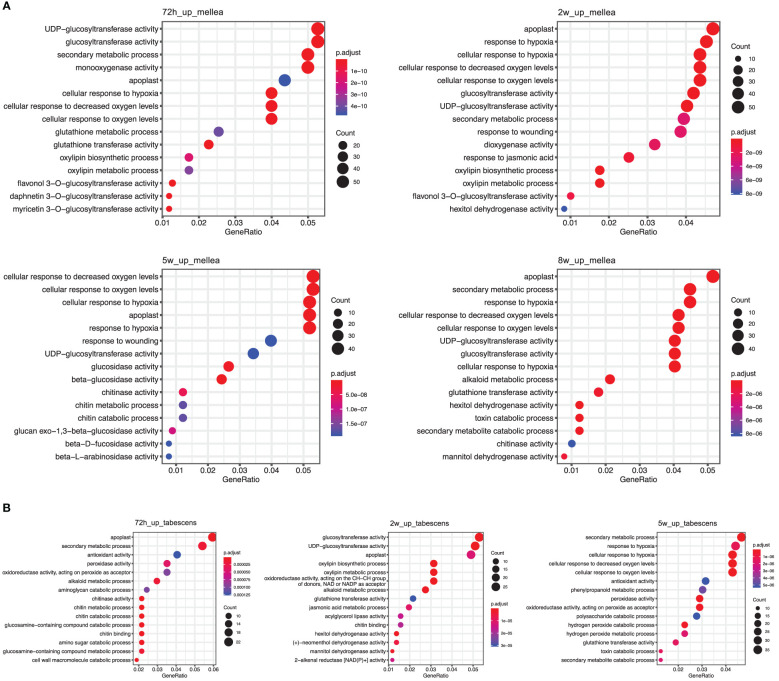
Gene Ontology (GO) term enrichment of differentially express genes in ‘MP-29’. **(A)** GO enrichment results of *A. mellea* treatment at four time points were visualized by dot plot. **(B)** GO enrichment results of *D. tabescens* treatment at three time points were visualized by dot plot.

To gain insight into the biological processes and gene functions involved in ARR resistance, we identified the most significantly enriched Gene Ontology (GO) terms at each time point in both *A. mellea* and *D. tabescens* treatments. For the *A. mellea* treatment, the results showed that most of the enriched GO terms were shared between time points (**Figure 4**). For example, at 72 hours post-inoculation, the most significantly enriched molecular function terms were “glucosyltransferase activity”, “monooxygenase activity”, and “glutathione transferase activity”, along with “secondary metabolic process”, “cellular response to hypoxia”, and “cellular response to oxygen levels” as the most enriched biological process terms. Additionally, “response to wounding” was only present at 2 weeks and 5 weeks. Compared to the *A. mellea* treatment, unique GO terms identified in the *D. tabescens* treatment, included “antioxidant activity”, “peroxidase activity”, and “dehydrogenase activity” as the enriched molecular function terms, and “cell wall macromolecule catabolic process”, “amino sugar catabolic process”, and “phenylpropanoid metabolic process” as the enriched biological process terms (**Figure 4**). The differences in enriched terms between treatments reinforce that ‘MP-29’ exhibited different responses to the two fungal infections. Collectively, these observations suggest possible molecular functions and involved biological processes of key genes underlying the ARR resistance.

### 3.5 Gene co-expression network analysis and the identification of hub genes

**Figure 5:**
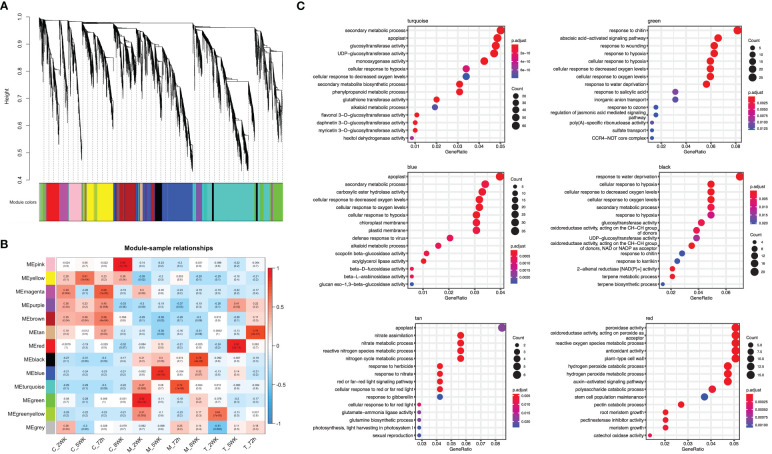
Weighted gene co-expression network analysis (WGCNA) of differentially expressed genes. **(A)** A hierarchical cluster dendrogram showing the co-expression modules. The genes were clustered based on dissimilarity measure. **(B)** Module-sample relationships: each row corresponds to a module and each column corresponds to a sample (treatment/timepoint). The coefficient of correlation and its corresponding P-value were presented in each box. **(C)** GO term enrichment for each WGCNA identified module in ‘MP-29’.

Weighted gene co-expression network analysis (WGCNA), to gain insight into the molecular mechanisms of ARR resistance, revealed 12 distinct modules, indicated with different colors in **Figure 5A**. Each module comprised clusters of genes with similar expression patterns. These modules can help explain biological processes and identify key genes associated with them. The number of DEGs ranged from 130 to 2,235 in each module. Additionally, seven modules (turquoise, green, blue, black, tan, green, yellow, and red) displayed a significantly high correlation with each infected sample, as shown in **Figure 5B**. Five hub genes were identified from each module based on their connectivity and significance (**Supplementary Table 3**).

**Figure 6:**
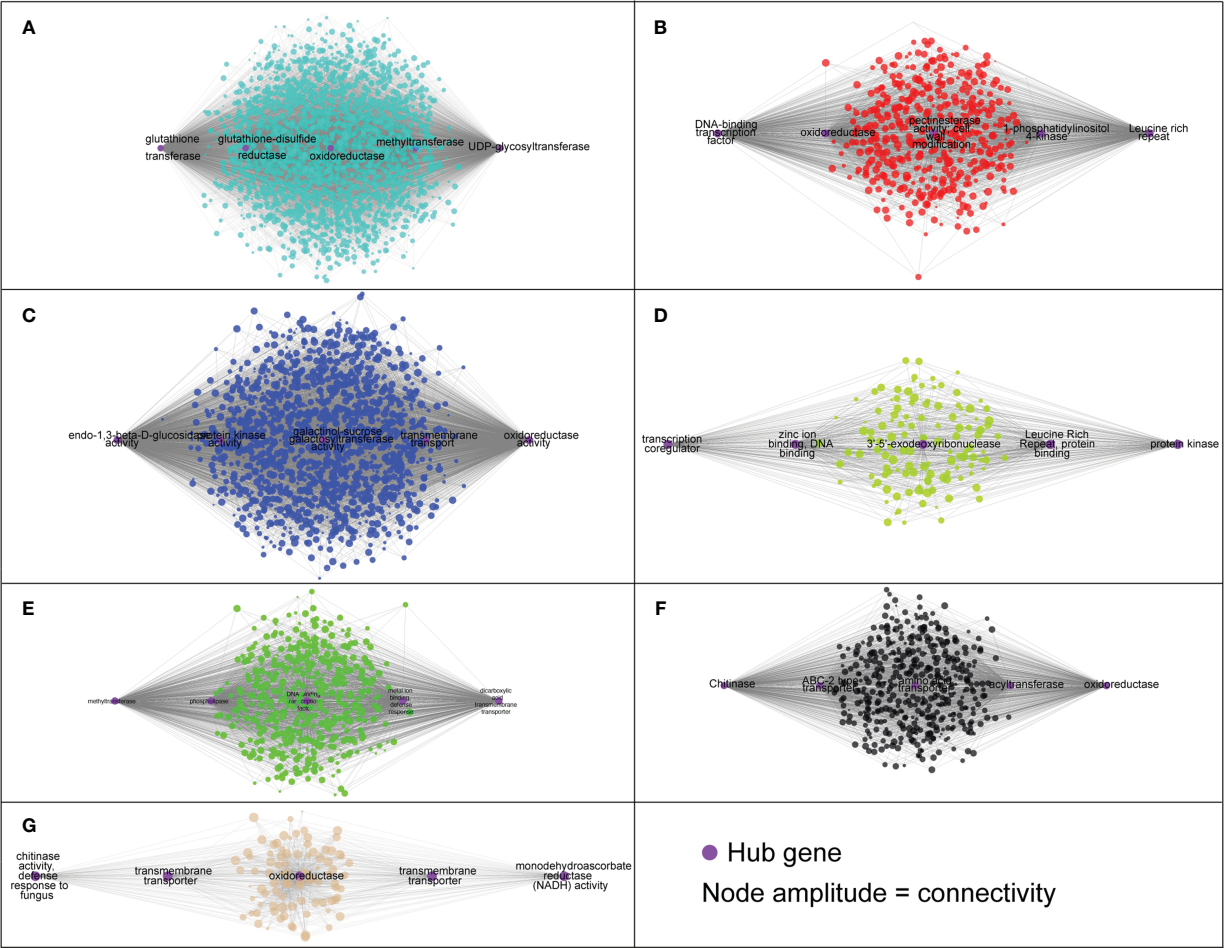
The hub genes identified in each module based on the co-expression analysis. **(A)** ‘turquoise’, **(B)** ‘red’, **(C)** ‘blue’, **(D)** ‘green yellow’, **(E)** ‘green’, **(F)** ‘black’ and **(G)** ‘tan’.

The module designated as “turquoise” is found to be significantly correlated with samples infected with *A. mellea* at 72 hours, with a correlation coefficient of 0.74. Analysis of the enriched GO terms within this module revealed enrichment for processes related to secondary metabolism and glucosyltransferase activity. Furthermore, the hub genes identified within this module have been previously found to play a role in detoxification, including genes encoding for enzymes such as UDP-glycosyltransferase, methyltransferase, oxidoreductase, glutathione-disulfide reductase, and glutathione transferase (**Figures 5C**, **6**, **Supplementary Table 3**). A separate module, designated as “green”, was found to be highly correlated with samples infected with *A. mellea* at 2 weeks, with a correlation coefficient of 0.92. This module was enriched for GO terms related to response to chitin, wounding, hypoxia, oxygen levels, water deprivation, and salicylic acid. The hub genes within this module include those encoding for a dicarboxylic acid transmembrane transporter, DNA-binding transcription factor, and defense response-related genes (**Figures 5C**, **6**, **Supplementary Table 3**).

The modules designated as “blue” and “black” were found to be significantly correlated with samples infected with *A. mellea* at 5 and 8 weeks, respectively. Analysis of the enriched GO terms within these modules revealed similarities to those identified in the previously discussed “turquoise” and “green” modules. Specifically, protein kinase and chitinase were identified as hub genes for the “blue” and “black” modules, respectively (**Figures 5C**, **6**, **Supplementary Table 3**). Additionally, three further modules were found to correspond to samples infected with *D. tabescens*. These include the “tan” module for samples at 72 hours, the “green yellow” module for samples at 2 weeks, and the “red” module for samples at 5 weeks. Analysis of these modules revealed unique GO terms, such as those related to nitrate metabolism and peroxidase activity. Notably, no GO terms were significantly enriched in the “green yellow” module. The only hub genes identified in the “green yellow” and “red” modules were leucine-rich repeat proteins (**Figures 5C**, **6**, **Supplementary Table 3**). Overall, these results provide insights into the molecular mechanisms underlying resistance to *A. mellea* and *D. tabescens*, as well as a gene list that may be useful for biomarker development.

### 3.6 Differential expression analysis in *Prunus cerasifera* (‘14-4’) and ‘Guardian®’

**Figure 7:**
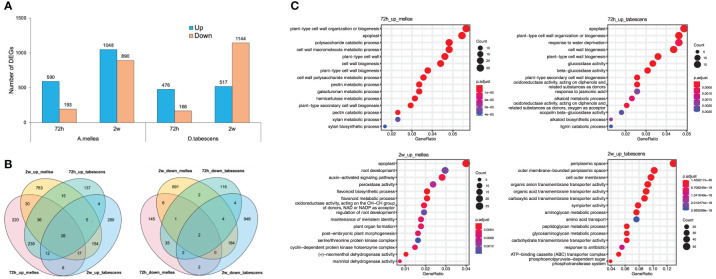
Differential transcriptomic responses to fungal infections in *Prunus cerasifera* (14-4). **(A)** The number of differentially expressed genes (P value < 0.01, log2(fold change) > 2) in each treatment/timepoint for ‘14-4’. **(B)** Venn diagrams comparing all DEGs between timepoints and treatments. **(C)** GO term enrichment of differentially express genes.

A differential expression analysis was conducted to examine the differences in resistance of *Prunus cerasifera* (‘14-4’) to *A. mellea* and *D. tabescens*. The analysis identified 783 and 642 differentially expressed genes (DEGs) for *A. mellea* and *D. tabescens* treatments at 72 hours, respectively (**Figure 7A**, **Supplementary File 2**). The number of DEGs increased at the subsequent time point of 2 weeks, with 1,938 DEGs for *A. mellea* and 1,661 DEGs for *D. tabescens*. The analysis indicated that more DEGs were up-regulated than down-regulated for all comparisons except for *D. tabescens* at 2 weeks. The majority of DEGs were unique between the two fungal treatments, demonstrating distinct responses of *P. cerasifera* to *A. mellea* and *D. tabescens* (**Figure 7B**). A total of 28 DEGs were consistently up-regulated across all treatments and time points (**Supplementary Table 4**). The GO enrichment analysis of ‘14-4’ showed an overrepresentation of plant cell wall-related terms, such as cell wall organization and biogenesis, cell wall macromolecule metabolism, pectin metabolism, and cell wall polysaccharide metabolism, at 72 hours for both treatments (**Figure 7C**, **Supplementary Table 5**). These results suggest a connection between the varying levels of ARR resistance in the two species. DEGs were also identified for the susceptible genotype ‘Guardian’® and the results were summarized in **Supplementary File 3**.

### 3.7 Ortholog analysis between different *Prunus* genotypes

An ortholog analysis was conducted to identify potential genes associated with ARR resistance in ‘MP-29’. This method was selected as it was hypothesized that the ARR resistance genes in ‘MP-29’ originated from plum rather than peach. As a result, transcripts present only in plum were of interest. Out of the 146,710 ‘MP-29’ transcripts assembled, 112,150 and 115,013 had orthologs in ‘Guardian®’ and ‘14-4’, respectively. The identification of these orthologs allowed us to compare the difference in gene expression across *Prunus* genotypes used in this study (**Supplementary File 4**). A total of 102,076 ‘MP-29’ transcripts were found to have orthologs identified in both ‘Guardian®’ and ‘14-4’. We observed that most orthologs differentially expressed in ‘MP-29’ have no difference in gene expression in ‘Guardian®’ and ‘14-4’ across time points and treatments. For example, 3,821 (97.5%) and 3748 (94.9%) orthologs out of 3,918 DEGs identified in ‘MP-29’ for *A. mellea* treatment at 72 hours exhibited no difference in gene expression in ‘Guardian®’ and ‘14-4’, respectively. These results not only explain the observed resistance in ‘MP-29’ compared to ‘Guardian®’ but the different resistant mechanisms between ‘MP-29’ and ‘14-4’.

**Figure 8:**
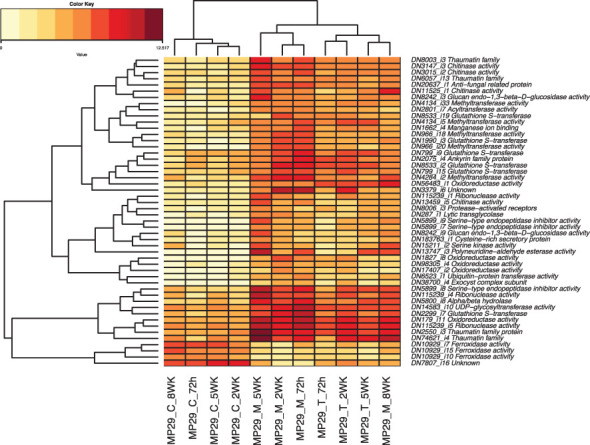
Heatmap of the 50 Differentially Expressed Genes (DEGs) that were consistently up- or down-regulated across different treatments and timepoints.

We identified a total of 405 transcripts were up- or down-regulated in ‘MP-29’ and ‘14-4’ at the same time but not significantly regulated in the ‘Guardian®’. GO enrichment analysis of these genes indicates that oxidoreductase activity, chitinase activity, response to jasmonic acid could play a role in the resistance to *Armillaria* since these terms were also highlighted in single species analysis (**Supplementary Figure 2**). The presence or absence of orthologs was examined for the 35 hub genes identified by network analysis and 50 common DEGs that were consistently up- or down-regulated across treatments and time points (**Figure 8**). Only two hub genes and six common DEGs were found to have orthologs presented in '14-4' but no orthologs presented in ‘Guardian®’(**Supplementary Tables 2, 3**). Two hub genes include a DNA-binding transcription factor (‘TRINITY_DN4194_c0_g1_i9’) and an oxidoreductase enzyme (‘TRINITY_DN25216_c0_g1_i2’). Six common DEGs include glutathione S-transferase (‘TRINITY_DN8533_c1_g1_i19’), oxidoreductase (‘TRINITY_DN17407_c0_g1_i2’), transglycolase (‘TRINITY_DN287_c0_g1_i1’) that have potential antifungal activity against *Armillaria* (**Figure 8**).

### 3.8 Expression analysis of disease resistance genes (RLK, RLP and NBS-LRR) between different *Prunus* genotypes

Based on transcriptome data, we have identified 1,219 transcripts of nucleotide-binding site, leucine-rich repeat (NBS-LRR or R genes, Pfam ID: PF00931), 92 transcripts of receptor-like kinases (RLK), and 340 transcripts of receptor-like proteins (RLP) in ‘MP-29’. Out of these, a total of 130 transcripts (107 R genes, 5 PLKs and 18 PLPs) exhibited differential expression for at least one treatment or time point (**Supplementary File 5**). However, no disease resistance gene was found to be consistently up- or down-regulated across all treatments and time points. Notably, R gene ‘TRINITY_DN317_c0_g1_i1’ was consistently downregulated at all time points for the *A. mellea* treatment, but not induced for *D. tabescens* treatment at all (**Supplementary Figure 3**). The Transcript ‘TRINITY_DN619_c0_g1_i1’ was expressed relatively high in control samples, and significantly downregulated in both fungal treatments quickly after 72 hours (**Supplementary File 5**). An induce R-gene (‘TRINITY_DN15385_c1_g1_i14’) was upregulated late in the co-culture, around 5 weeks, in both fungal treatments. By comparing the expression of disease resistance genes in different *Prunus* genotypes using ortholog analysis, we observed that almost all disease resistance genes that were up- or down-regulated in ‘MP-29’ were not significantly regulated in ‘Guardian®’ and ‘14-4’ (**Supplementary File 4**). These results suggest that disease resistance genes have a role in *Armillaria* resistance and further reinforce the existence of different resistant mechanisms among *Prunus* genotypes.

## 4 Discussion

Identification of genetic resistance to ARR in peach rootstocks is of great importance for the management and control of this destructive disease. The use of gene expression analysis to identify differentially expressed genes (DEGs) in peach rootstocks that are resistant to the fungus can provide valuable insights into the molecular mechanisms underlying ARR resistance. This information can then be used to develop strategies for improving the resistance of peach orchards to the disease. Furthermore, the identification of hub genes and DEGs that are consistently up- or down-regulated across different treatments and time points can provide a gene list that may be useful for biomarker development. Additionally, ortholog analysis between species can further filter the candidate genes related to ARR resistance, specifically those that originated from plum rather than peach, as it was shown that the ARR resistance comes from plum (Beckman et al., 2012). This knowledge could help in the development of new resistant cultivars, which would be of great benefit to the peach industry.

In our *in vitro* co-culture experiments, we challenged three *Prunus* genotypes, two rootstocks and one *P. cerasifera* accession, with two ARR species and were able to collect detailed and accurate phenotypic data over the course of the study (Adelberg et al., 2021). This was achieved by maintaining optimal control over the experimental environment, including light, temperature, growth media components, and isolation from any potential contaminants, such as endophytes. One particularly challenging component to the experiment was the harvesting and manipulation of infected plant roots, as the amount of biomass was limited. One major challenge was the presence of polysaccharides, polyphenols, and other contaminants that can interfere with the extraction and purification of RNA, leading to poor yields and low quality of the final RNA product. The presence of fungal pathogens can also lead to the degradation of the plant’s RNA, resulting in low yield and poor quality of the RNA, which was observed in ‘14-4’ and several ‘Guardian®’ samples over time. Additionally, the fungal infection and/or direct competition for nutrient resources between the fungus and the plant can lead to changes in the metabolic activity of the plant (stress metabolism), which can further complicate the extraction and purification of RNA and reveal genetic signatures not directly related to disease.

Our study observed differential responses to the two fungi, *A. mellea* and *D. tabescens*, in the ARR resistant rootstock and plum accession, ‘MP-29’ and ‘14-4’. Notably, ‘14-4’ exhibited substantial resistance to *D. tabescens*, while ‘MP-29’ showed a higher level of resistance to *A. mellea* (**Figure 1**). In contrast, the ‘Guardian®’ (ARR susceptible) rootstock showed a decline in plant health around 2 weeks, and rapidly worsened thereafter. These results suggest that each genotype may have a unique mechanism of resistance to the fungi. Additionally, these observations indicate that the two fungi may have unique infection dynamics.

To dissect these observations at the genetic level, we used high-resolution transcriptomics from each of the three genotypes over an infection time course. The BUSCO analysis of single-copy gene ortholog assessments of the *de novo* transcriptome assemblies indicate a high level of completeness (>94% for each) providing a high-quality dataset. An analysis of diversity based on gene expression using principal components in ‘MP-29’ revealed separation of the fungal treatments primarily by the first principal component. The second principal component showed differences based on the time post-inoculation, indicating that the treatment response was the more significant variable (**Figure 2**). Samples from 72h and 2 weeks clustered for each treatment on the second principal coordinate, which suggests that the signaling and initiation of the resistant response (at the gene level) takes place early (within the first 3 days upon infection).

Pairwise gene expression analysis showed distinct transcriptional responses in ‘MP-29’ to *A. mellea* and *D. tabescens*, suggesting different defense mechanisms may be involved (**Figure 3**). The gene expression profiles support the diversity observed in the principal component analysis (**Figure 2**), indicating a swift genetic response in ‘MP-29’ to each pathogen. There were 46 genes that were upregulated in ‘MP-29’ in response to both *A. mellea and D. tabescens* that were common across time points and treatments. These genes include Glutathione S-Transferases (GSTs), hydrolases, and ribonucleases. Glutathione S-transferase genes play a critical role in plant resistance to fungal pathogens. GSTs are involved in detoxifying harmful substances produced by fungi, including phytotoxins, and they can also act as scavengers of reactive oxygen species generated during the plant’s defense response (Gullner et al., 2018; Wahibah et al., 2018). Studies have demonstrated the significance of GSTs in regulating plant defense against various fungal pathogens and abiotic stressors. For instance, the over-expression of a particular GST gene in *Brassica napus* was shown to improve its resistance to powdery mildew (Mikhaylova et al., 2021). In transgenic tobacco plants expressing a GST from *Pyrus pyrifolia*, improved tolerance to abiotic stress was observed (Liu et al., 2013). Another study demonstrated that transgenic tobacco plants overexpressing an alfalfa GST exhibited improved saline tolerance (Du et al., 2019). Plant hydrolytic enzymes also play an important role in plant defense against fungal pathogens. These enzymes break down fungal cell walls and help to prevent pathogen attachment and penetration into the plant. Some examples of hydrolytic enzymes involved in plant defense include chitinases (Punja and Zhang, 1993) and beta-1,3-glucanases (Balasubramanian et al., 2012). RNA degrading enzymes have also been implicated in response to pathogen attack and as a component of host resistance (Singh et al., 2020).

Gene set enrichment analysis revealed significant enrichment of genes in biological processes, molecular functions, and cellular components (**Figure 4**). The 72-hour post-inoculation timepoint showed the highest significance, with the enriched terms being “glucosyltransferase activity”, “monooxygenase activity”, and “glutathione transferase activity”, and biological processes such as “secondary metabolic process”, “cellular response to hypoxia”, and “cellular response to oxygen levels.” In contrast, the *D. tabescens* challenge revealed enriched molecular function terms such as “antioxidant activity”, “peroxidase activity”, and “dehydrogenase activity”, as well as enriched cellular components including “cell wall macromolecule catabolic process”, “amino sugar catabolic process”, and “phenylpropanoid metabolic process.” Expression of genes and biochemical pathways in these categories indicate sophisticated genetic response that includes activation of enzymatic pathways, defense related genes, and mechanisms that may activate cell wall biosynthesis leading to a physical barrier of defense. These results suggest that ‘MP-29’ exhibited distinct responses to the two fungal pathogens and indicate potential mechanisms of host defense. Upon directed analysis of disease resistance genes (R-genes) during fungal co-culture, we observed an interesting pattern of expression. Although a total of 130 transcripts exhibited differential expression for at least one treatment or timepoint, we did not observe any apparent signatures of R-gene upregulation specifically at early timepoints. However, we did detect transcriptional signals at later timepoints (after 2 weeks), which could potentially indicate the activation of effector-triggered immunity.

The construction of a gene co-expression network is a valuable technique for deciphering relationships among genes and their functions from gene expression data. This process maps the interdependence of multiple genes, represented as nodes, by connecting them with edges reflecting the correlation strength and direction. The network visually represents gene interactions and unveils complex regulatory relationships, including those implicated in specific biological processes or responses. Hub genes, as key nodes, can have a significant impact on gene regulation and act as central regulators of gene expression. The network is further composed of modules, clusters of highly interconnected genes that may indicate functional units or biological processes. The results indicated that seven of the twelve modules exhibited a strong correlation with infected samples (**Figure 5B**). The turquoise module, comprised of hub genes associated with plant defense against pathogens such as glutathione transferase, glutathione-disulfide reductase, oxidoreductase, methyltransferase, and UDP-glycosyltransferase, demonstrated significant correlation with the ‘MP-29’ response at 72 hours post-challenge with *A. mellea* (**Figure 6A**). Glutathione-disulfide reductase (GSR) is crucial in plant antifungal defense by reducing glutathione disulfides to glutathione, preserving its reduced state, and enabling its function as an antioxidant, thereby protecting the plant against oxidative stress during fungal attack (Zechmann, 2020). Oxidoreductases can alter the redox state of plant cells, triggering the expression of defense-related genes, thereby enhancing the plant’s resistance to fungal attacks (Blee, 1998). Histone methyltransferase activity has been demonstrated to play a vital role in plant defense against fungal pathogens by regulating a subset of genes within the jasmonic acid (JA) and/or ethylene signaling pathway (Berr et al., 2010). Methylation also allows the plant to retain a memory of previous encounters with pathogens, resulting in stronger and quicker defense mechanisms during future infections. UDP glycosyltransferases, a multigenic and diverse superfamily of enzymes, are involved in the synthesis and modification of plant secondary metabolites, including phytohormones and phytoalexins, which play crucial roles in plant defense against biotic and abiotic stress, including fungal pathogens (Gachon et al., 2005). These enzymes modify the structure of phytohormones such as salicylic acid and jasmonic acid, which serve as central signaling molecules in plant defense response. For instance, UDPs can modify SA to produce novel compounds that modulate SA signaling, leading to increased defense responses (von Saint Paul et al., 2011). They also participate in the biosynthesis of phytoalexins, toxic compounds produced by plants in response to pathogen attack, serving as direct protection against fungal infections (Reim et al., 2021).

Other hub genes present in multiple modules include various transcription factors, oxidoreductases, Leucine Rich Repeats (LRRs), chitinases, and various transporters (**Figure 6**). Notably, Chitinases are enzymes that play a key role in plant defense against fungal pathogens. They degrade chitin, a component of fungal cell walls, causing damage and weakening the fungal structure, which results in reduced fungal growth and increased plant resistance. Additionally, the production of chitinases can trigger the expression of other defense-related genes, leading to a more robust plant defense response. Chitinases are considered hub genes, as they are commonly present in multiple gene modules involved in plant defense (Wang et al., 2020). The expression of chitinases has been linked to salicylic acid and jasmonic acid signaling pathways, two central signaling molecules in plant defense response. Overall, chitinases are important components of the plant’s defense mechanism against fungal pathogens.

Our transcriptomic analysis uncovers intricate molecular processes governing the *Armillaria* root rot (ARR) response in *Prunus* spp. Our findings show that each genotype demonstrated distinct responses to infections caused by *A. mellea* and *D. tabescens*, as reflected by plant health performance and transcriptional alterations. Additionally, we observed variations in ARR resistance among different genotypes when infected with the same *Armillaria* fungi. Importantly, our study identified multiple components contributing to ARR resistance, including detoxifying enzymes (e.g., GSTs, UDP-glycosyltransferases, oxidoreductases), Leucine Rich Repeats (LRRs), chitinases, and various transcription factors. These results offer not only new insights into the molecular regulation of ARR resistance, but also serve as a valuable resource for the improvement of ARR resistance in *Prunus* rootstocks through breeding.

## Data availability statement

The original contributions presented in the study are publicly available. This data can be found here: https://www.ncbi.nlm.nih.gov/bioproject/PRJNA930771/.

## Author contributions

CS, KG, JA, GS, GR conceived, designed, and acquired funding the study. LC analyzed data and wrote the first draft of the manuscript. AC and ZL analyzed data and edited the manuscript. JA and JN-A prepared and maintained *in vitro* cultures, collected, and analyzed data, and edited the manuscript. CS, KG, JA, GS, GR edited the manuscript. All authors approve of the final version.
