# Supplementary material for: Transcriptomics reveal the genetic coordination of early defense to Armillaria root rot (ARR) in Prunus spp
Source: Front Plant Sci. 2023 Jun 2;14:1181153. doi: 10.3389/fpls.2023.1181153 (PMC10274510; doi:10.3389/fpls.2023.1181153)
Supplement: Supplementary file 1 [file DataSheet_1.docx]

Supplementary Figure 1. Differential transcriptomic responses to fungal infections. Venn diagrams comparing both up- and down-regulated DEGs between treatments at the same timepoint in ‘MP-29’.

Supplementary Figure 2. GO term enrichment of genes which were up- or down-regulated in ‘MP-29’ and ‘14-4’ at the same time but not significantly induced in the ‘Guardian®’ across different time points and treatments.

Supplementary Figure 3. Heatmap of the 130 R genes (NBS-LRR, RPL, RPK) that were differentially expressed across different treatments and timepoints.

Supplementary Table 1. Summary of the sequencing reads used in this study.

| Sample | Raw reads | Raw data(G) | Q20(%) | Q30(%) | GC(%) | Clean_1(quality control) | Clean_1(%) | Clean_2(decontamination) | Clean_2(%) |
| --- | --- | --- | --- | --- | --- | --- | --- | --- | --- |
| Guard_C_72h1 | 102,067,386 | 15,310,107,900 | 98.38 | 95.33 | 45.82 | 96,581,666 | 94.63 | 96,547,258 | 94.59 |
| Guard_C_72h2 | 100,122,954 | 15,018,443,100 | 98.35 | 95.24 | 45.79 | 94,445,160 | 94.33 | 94,435,558 | 94.32 |
| Guard_C_72h3 | 86,835,840 | 13,025,376,000 | 98.38 | 95.37 | 45.91 | 81,953,972 | 94.38 | 81,922,704 | 94.34 |
| Guard_C_2WK1 | 115,365,382 | 17,304,807,300 | 98.41 | 95.45 | 45.86 | 109,067,256 | 94.54 | 109,055,266 | 94.53 |
| Guard_C_2WK2 | 102,259,958 | 15,338,993,700 | 98.42 | 95.44 | 45.95 | 95,656,030 | 93.54 | 95,638,258 | 93.52 |
| Guard_C_2WK3 | 106,476,906 | 15,971,535,900 | 98.34 | 95.24 | 45.88 | 100,411,852 | 94.30 | 100,384,394 | 94.28 |
| Guard_C_5WK1 | 97,130,232 | 14,569,534,800 | 98.26 | 95.03 | 45.42 | 91,109,930 | 93.80 | 91,100,648 | 93.79 |
| Guard_C_5WK2 | 101,554,362 | 15,233,154,300 | 98.25 | 94.97 | 45.47 | 95,482,880 | 94.02 | 95,471,172 | 94.01 |
| Guard_C_5WK3 | 99,870,364 | 14,980,554,600 | 98.26 | 95.04 | 45.74 | 93,758,802 | 93.88 | 93,745,082 | 93.87 |
| Guard_C_8WK1 | 81,836,312 | 12,275,446,800 | 97.52 | 93.57 | 45.85 | 75,334,576 | 92.06 | 75,297,362 | 92.01 |
| Guard_C_8WK2 | 91,372,616 | 13,705,892,400 | 97.29 | 92.99 | 45.86 | 82,722,032 | 90.53 | 82,683,598 | 90.49 |
| Guard_C_8WK3 | 83,269,514 | 12,490,427,100 | 97.61 | 93.68 | 45.85 | 76,095,110 | 91.38 | 76,059,464 | 91.34 |
| Guard_M_72h1 | 86,030,866 | 12,904,629,900 | 98.51 | 95.66 | 45.97 | 81,417,544 | 94.64 | 80,486,560 | 93.56 |
| Guard_M_72h2 | 101,640,834 | 15,246,125,100 | 98.43 | 95.44 | 46.05 | 95,876,382 | 94.33 | 94,713,942 | 93.18 |
| Guard_M_72h3 | 91,019,632 | 13,652,944,800 | 98.40 | 95.46 | 45.90 | 85,609,992 | 94.06 | 84,605,452 | 92.95 |
| Guard_M_2WK1 | 94,351,082 | 14,152,662,300 | 98.76 | 96.22 | 50.37 | 89,962,568 | 95.35 | 25,480,956 | 27.01 |
| Guard_M_2WK2 | 121,646,068 | 18,246,910,200 | 98.71 | 96.04 | 50.16 | 116,323,584 | 95.62 | 35,217,422 | 28.95 |
| Guard_M_2WK3 | 103,212,194 | 15,481,829,100 | 98.71 | 96.04 | 49.86 | 98,682,920 | 95.61 | 31,663,080 | 30.68 |
| Guard_T_72h1 | 84,185,038 | 12,627,755,700 | 98.27 | 95.16 | 44.36 | 78,002,504 | 92.66 | 77,231,748 | 91.74 |
| Guard_T_72h2 | 92,761,450 | 13,914,217,500 | 98.37 | 95.43 | 45.93 | 87,364,444 | 94.18 | 86,662,614 | 93.43 |
| Guard_T_72h3 | 99,617,066 | 14,942,559,900 | 98.28 | 95.34 | 45.93 | 93,505,620 | 93.87 | 92,671,778 | 93.03 |
| Guard_T_5WK1 | 94,761,400 | 14,214,210,000 | 98.22 | 95.03 | 47.95 | 88,336,860 | 93.22 | 58,139,480 | 61.35 |
| Guard_T_5WK2 | 109,313,416 | 16,397,012,400 | 98.24 | 95.02 | 46.95 | 102,432,514 | 93.71 | 79,202,666 | 72.45 |
| Guard_T_5WK3 | 93,404,096 | 14,010,614,400 | 98.21 | 94.89 | 46.70 | 87,638,918 | 93.83 | 70,260,926 | 75.22 |
| MP29_C_72h1 | 117,450,478 | 17,617,571,700 | 98.52 | 95.59 | 45.63 | 111,784,230 | 95.18 | 111,732,004 | 95.13 |
| MP29_C_72h2 | 96,820,480 | 14,523,072,000 | 98.52 | 95.62 | 45.73 | 92,010,028 | 95.03 | 91,973,388 | 94.99 |
| MP29_C_72h3 | 108,066,730 | 16,210,009,500 | 98.42 | 95.31 | 45.76 | 102,329,250 | 94.69 | 102,283,778 | 94.65 |
| MP29_C_2WK1 | 122,250,394 | 18,337,559,100 | 98.33 | 95.10 | 45.61 | 115,428,616 | 94.42 | 115,408,394 | 94.40 |
| MP29_C_2WK2 | 102,355,656 | 15,353,348,400 | 98.54 | 95.62 | 45.54 | 97,455,528 | 95.21 | 97,442,482 | 95.20 |
| MP29_C_2WK3 | 112,634,594 | 16,895,189,100 | 98.50 | 95.51 | 45.50 | 107,164,374 | 95.14 | 107,121,918 | 95.11 |
| MP29_C_5WK1 | 98,541,696 | 14,781,254,400 | 98.31 | 95.11 | 45.56 | 92,333,148 | 93.70 | 92,303,946 | 93.67 |
| MP29_C_5WK2 | 85,503,100 | 12,825,465,000 | 98.35 | 95.29 | 45.79 | 80,250,120 | 93.86 | 80,226,750 | 93.83 |
| MP29_C_5WK3 | 95,449,448 | 14,317,417,200 | 98.17 | 94.75 | 45.63 | 89,596,734 | 93.87 | 89,578,726 | 93.85 |
| MP29_C_8WK1 | 86,476,826 | 12,971,523,900 | 97.53 | 93.55 | 45.87 | 78,976,566 | 91.33 | 78,943,250 | 91.29 |
| MP29_C_8WK2 | 87,813,722 | 13,172,058,300 | 97.28 | 92.98 | 45.86 | 80,184,058 | 91.31 | 80,143,072 | 91.26 |
| MP29_C_8WK3 | 90,002,156 | 13,500,323,400 | 97.64 | 93.76 | 45.85 | 82,682,938 | 91.87 | 82,665,934 | 91.85 |
| MP29_M_72h1 | 105,702,146 | 15,855,321,900 | 98.51 | 95.61 | 46.12 | 100,197,418 | 94.79 | 95,864,040 | 90.69 |
| MP29_M_72h2 | 111,310,306 | 16,696,545,900 | 98.47 | 95.53 | 46.07 | 105,565,698 | 94.84 | 100,876,390 | 90.63 |
| MP29_M_72h3 | 95,844,068 | 14,376,610,200 | 98.35 | 95.24 | 46.10 | 90,463,198 | 94.39 | 86,357,400 | 90.10 |
| MP29_M_2WK1 | 87,504,408 | 13,125,661,200 | 98.55 | 95.76 | 47.81 | 83,234,118 | 95.12 | 59,497,700 | 67.99 |
| MP29_M_2WK2 | 100,564,416 | 15,084,662,400 | 98.45 | 95.52 | 47.80 | 95,202,620 | 94.67 | 66,333,328 | 65.96 |
| MP29_M_2WK3 | 104,704,432 | 15,705,664,800 | 98.44 | 95.47 | 47.50 | 98,600,118 | 94.17 | 72,648,904 | 69.38 |
| MP29_M_5WK1 | 83,789,658 | 12,568,448,700 | 98.30 | 95.19 | 46.30 | 78,215,046 | 93.35 | 71,720,086 | 85.60 |
| MP29_M_5WK2 | 108,341,348 | 16,251,202,200 | 98.40 | 95.35 | 46.26 | 102,662,584 | 94.76 | 95,354,054 | 88.01 |
| MP29_M_5WK3 | 88,559,790 | 13,283,968,500 | 98.30 | 95.10 | 46.10 | 83,764,068 | 94.58 | 78,127,854 | 88.22 |
| MP29_M_8WK1 | 92,549,998 | 13,882,499,700 | 97.88 | 94.17 | 46.60 | 85,012,194 | 91.86 | 77,414,722 | 83.65 |
| MP29_M_8WK2 | 84,137,434 | 12,620,615,100 | 98.01 | 94.52 | 46.42 | 77,501,326 | 92.11 | 70,871,314 | 84.23 |
| MP29_M_8WK3 | 82,762,934 | 12,414,440,100 | 97.80 | 94.15 | 45.60 | 75,376,940 | 91.08 | 68,770,278 | 83.09 |
| MP29_T_72h1 | 97,059,404 | 14,558,910,600 | 98.45 | 95.52 | 45.76 | 91,605,560 | 94.38 | 91,478,164 | 94.25 |
| MP29_T_72h2 | 104,724,526 | 15,708,678,900 | 98.44 | 95.50 | 45.80 | 98,857,038 | 94.40 | 98,738,446 | 94.28 |
| MP29_T_72h3 | 98,628,082 | 14,794,212,300 | 98.41 | 95.48 | 45.73 | 93,020,630 | 94.31 | 92,912,854 | 94.21 |
| MP29_T_2WK1 | 104,479,614 | 15,671,942,100 | 98.61 | 95.84 | 46.52 | 99,662,680 | 95.39 | 83,535,392 | 79.95 |
| MP29_T_2WK2 | 89,823,054 | 13,473,458,100 | 98.61 | 95.92 | 45.90 | 83,774,830 | 93.27 | 69,913,858 | 77.84 |
| MP29_T_2WK3 | 86,695,440 | 13,004,316,000 | 98.61 | 95.91 | 46.58 | 82,084,628 | 94.68 | 68,068,594 | 78.51 |
| MP29_T_5WK1 | 84,509,984 | 12,676,497,600 | 98.27 | 95.12 | 45.85 | 79,225,480 | 93.75 | 76,756,958 | 90.83 |
| MP29_T_5WK2 | 100,050,802 | 15,007,620,300 | 98.29 | 95.10 | 45.71 | 94,023,064 | 93.98 | 91,570,008 | 91.52 |
| MP29_T_5WK3 | 109,057,554 | 16,358,633,100 | 98.25 | 94.96 | 45.83 | 102,580,884 | 94.06 | 99,865,392 | 91.57 |
| s14_4_C_72h1 | 93,228,744 | 13,984,311,600 | 98.56 | 95.79 | 44.32 | 87,683,854 | 94.05 | 87,652,100 | 94.02 |
| s14_4_C_72h2 | 97,161,040 | 14,574,156,000 | 98.57 | 95.70 | 45.34 | 92,503,602 | 95.21 | 92,475,158 | 95.18 |
| s14_4_C_72h3 | 100,878,950 | 15,131,842,500 | 98.55 | 95.75 | 45.03 | 95,618,402 | 94.79 | 95,592,208 | 94.76 |
| s14_4_C_2WK1 | 110,064,282 | 16,509,642,300 | 98.35 | 95.21 | 45.51 | 103,512,234 | 94.05 | 103,477,544 | 94.02 |
| s14_4_C_2WK2 | 111,805,544 | 16,770,831,600 | 97.61 | 93.35 | 45.28 | 103,377,422 | 92.46 | 103,346,896 | 92.43 |
| s14_4_C_2WK3 | 97,254,732 | 14,588,209,800 | 98.35 | 95.22 | 45.48 | 91,312,962 | 93.89 | 91,285,168 | 93.86 |
| s14_4_M_72h1 | 117,053,460 | 17,558,019,000 | 98.52 | 95.59 | 45.43 | 110,721,038 | 94.59 | 110,609,332 | 94.49 |
| s14_4_M_72h2 | 113,402,610 | 17,010,391,500 | 98.41 | 95.34 | 45.44 | 107,333,604 | 94.65 | 107,227,362 | 94.55 |
| s14_4_M_72h3 | 96,794,956 | 14,519,243,400 | 98.58 | 95.71 | 45.35 | 91,849,426 | 94.89 | 91,751,872 | 94.79 |
| s14_4_M_2WK1 | 95,252,828 | 14,287,924,200 | 98.42 | 95.38 | 48.34 | 90,013,086 | 94.50 | 56,831,734 | 59.66 |
| s14_4_M_2WK2 | 96,446,676 | 14,467,001,400 | 98.30 | 95.09 | 47.58 | 90,761,890 | 94.11 | 62,068,072 | 64.35 |
| s14_4_M_2WK3 | 86,857,650 | 13,028,647,500 | 98.34 | 95.15 | 47.90 | 81,923,548 | 94.32 | 54,617,448 | 62.88 |
| s14_4_T_72h1 | 101,635,012 | 15,245,251,800 | 98.15 | 94.64 | 45.59 | 94,657,628 | 93.13 | 94,457,670 | 92.94 |
| s14_4_T_72h2 | 94,697,134 | 14,204,570,100 | 98.32 | 95.09 | 45.65 | 79,268,596 | 83.71 | 79,135,580 | 83.57 |
| s14_4_T_72h3 | 91,905,914 | 13,785,887,100 | 98.50 | 95.52 | 45.57 | 87,349,408 | 95.04 | 87,179,274 | 94.86 |
| s14_4_T_2WK1 | 93,872,232 | 14,080,834,800 | 98.32 | 95.18 | 45.76 | 88,152,436 | 93.91 | 88,079,528 | 93.83 |
| s14_4_T_2WK2 | 82,647,268 | 12,397,090,200 | 98.38 | 95.31 | 44.74 | 76,700,972 | 92.81 | 76,655,584 | 92.75 |
| s14_4_T_2WK3 | 122,761,648 | 18,414,247,200 | 98.23 | 94.99 | 45.79 | 114,790,128 | 93.51 | 114,668,482 | 93.41 |

Supplementary Table 2. Summary of the 50 consistently up- (46) or down- (4) regulated genes across different treatments and timepoints in ‘MP-29’.

| ID | If ortholog to Guardian | If ortholog to 14-4 | Annotation |
| --- | --- | --- | --- |
| TRINITY_DN6057_c0_g1_i13 | No | Yes | DN6057_i13 Thaumatin family |
| TRINITY_DN14583_c0_g1_i10 | Yes | No | DN14583_i10 UDP-glycosyltransferase activity |
| TRINITY_DN8006_c0_g1_i3 | Yes | Yes | DN8006_i3 Protease-activated receptors |
| TRINITY_DN6523_c5_g1_i1 | Yes | Yes | DN6523_i1 Ubiquitin-protein transferase activity |
| TRINITY_DN3379_c0_g1_i6 | Yes | Yes | DN3379_i6 Unknown |
| TRINITY_DN8533_c1_g1_i19 | No | Yes | DN8533_i19 Glutathione S-transferase |
| TRINITY_DN8533_c1_g1_i2 | No | No | DN8533_i2 Glutathione S-transferase |
| TRINITY_DN8003_c0_g1_i3 | Yes | Yes | DN8003_i3 Thaumatin family |
| TRINITY_DN17407_c0_g1_i2 | No | Yes | DN17407_i2 Oxidoreductase activity |
| TRINITY_DN56483_c0_g1_i1 | Yes | Yes | DN56483_i1 Oxidoreductase activity |
| TRINITY_DN799_c0_g1_i9 | Yes | Yes | DN799_i9 Glutathione S-transferase |
| TRINITY_DN15211_c0_g1_i2 | Yes | Yes | DN15211_i2 Serine kinase activity |
| TRINITY_DN3147_c2_g1_i3 | Yes | No | DN3147_i3 Chitinase activity |
| TRINITY_DN3015_c0_g2_i2 | Yes | Yes | DN3015_i2 Chitinase activity |
| TRINITY_DN183763_c0_g1_i1 | Yes | Yes | DN183763_i1 Cysteine-rich secretory protein |
| TRINITY_DN38700_c0_g1_i4 | Yes | Yes | DN38700_i4 Exocyst complex subunit |
| TRINITY_DN115239_c0_g1_i4 | Yes | Yes | DN115239_i4 Ribonuclease activity |
| TRINITY_DN287_c0_g1_i1 | No | Yes | DN287_i1 Lytic transglycolase |
| TRINITY_DN799_c0_g1_i15 | Yes | Yes | DN799_i15 Glutathione S-transferase |
| TRINITY_DN115239_c0_g1_i1 | Yes | Yes | DN115239_i1 Ribonuclease activity |
| TRINITY_DN1662_c0_g1_i4 | Yes | No | DN1662_i4 Manganese ion binding |
| TRINITY_DN74621_c0_g1_i4 | No | No | DN74621_i4 Thaumatin family |
| TRINITY_DN5899_c0_g1_i8 | Yes | Yes | DN5899_i8 Serine-type endopeptidase inhibitor activity |
| TRINITY_DN179_c0_g1_i11 | Yes | Yes | DN179_i11 Oxidoreductase activity |
| TRINITY_DN8242_c0_g1_i9 | Yes | Yes | DN8242_i9 Glucan endo-1,3-beta-D-glucosidase activity |
| TRINITY_DN1990_c0_g1_i3 | Yes | Yes | DN1990_i3 Glutathione S-transferase |
| TRINITY_DN11525_c0_g1_i1 | Yes | Yes | DN11525_i1 Chitinase activity |
| TRINITY_DN10929_c0_g1_i7 | Yes | Yes | DN10929_i7 Ferroxidase activity |
| TRINITY_DN13459_c0_g1_i5 | Yes | Yes | DN13459_i5 Chitinase activity |
| TRINITY_DN5899_c0_g1_i9 | Yes | Yes | DN5899_i9 Serine-type endopeptidase inhibitor activity |
| TRINITY_DN4134_c0_g1_i5 | Yes | Yes | DN4134_i5 Methyltransferase activity |
| TRINITY_DN115239_c0_g1_i5 | Yes | Yes | DN115239_i5 Ribonuclease activity |
| TRINITY_DN2075_c2_g1_i4 | No | Yes | DN2075_i4 Ankyrin family protein |
| TRINITY_DN5899_c0_g1_i7 | Yes | Yes | DN5899_i7 Serine-type endopeptidase inhibitor activity |
| TRINITY_DN2550_c1_g2_i3 | Yes | Yes | DN2550_i3 Thaumatin family protein |
| TRINITY_DN966_c0_g1_i18 | Yes | Yes | DN966_i18 Methyltransferase activity |
| TRINITY_DN98305_c0_g1_i4 | No | No | DN98305_i4 Oxidoreductase activity |
| TRINITY_DN1827_c4_g1_i8 | Yes | Yes | DN1827_i8 Oxidoreductase activity |
| TRINITY_DN2299_c0_g1_i7 | Yes | No | DN2299_i7 Glutathione S-transferase |
| TRINITY_DN7807_c1_g1_i16 | Yes | Yes | DN7807_i16 Unknown |
| TRINITY_DN4134_c0_g1_i33 | Yes | Yes | DN4134_i33 Methyltransferase activity |
| TRINITY_DN4284_c0_g1_i2 | Yes | Yes | DN4284_i2 Methyltransferase activity |
| TRINITY_DN2801_c0_g1_i7 | No | Yes | DN2801_i7 Acyltransferase activity |
| TRINITY_DN10929_c0_g1_i10 | Yes | Yes | DN10929_i10 Ferroxidase activity |
| TRINITY_DN966_c0_g1_i20 | Yes | Yes | DN966_i20 Methyltransferase activity |
| TRINITY_DN8242_c0_g1_i3 | Yes | Yes | DN8242_i3 Glucan endo-1,3-beta-D-glucosidase activity |
| TRINITY_DN10929_c0_g1_i15 | Yes | Yes | DN10929_i15 Ferroxidase activity |
| TRINITY_DN13747_c0_g1_i3 | No | No | DN13747_i3 Polyneuridine-aldehyde esterase activity |
| TRINITY_DN5800_c0_g2_i8 | Yes | Yes | DN5800_i8 Alpha/beta hydrolase |
| TRINITY_DN20637_c0_g1_i1 | Yes | Yes | DN20637_i1 Anti-fungal related protein |

Supplementary Table 3. Summary of the hub genes identified in each module for ‘MP-29’.

| Module | Gene | Annotation | If ortholog to Guardian | If ortholog to  14-4 |
| --- | --- | --- | --- | --- |
| Turquoise | TRINITY_DN14583_c0_g1_i10 | UDP-glycosyltransferase activity | Yes | No |
|  | TRINITY_DN966_c0_g1_i12 | methyltransferase activity | Yes | Yes |
|  | TRINITY_DN3814_c2_g1_i2 | oxidoreductase activity | Yes | Yes |
|  | TRINITY_DN2453_c0_g1_i11 | glutathione-disulfide reductase activity | No | No |
|  | TRINITY_DN1990_c0_g1_i3 | glutathione transferase activity | Yes | Yes |
| Green | TRINITY_DN4753_c0_g1_i2 | dicarboxylic acid transmembrane transporter activity | Yes | Yes |
|  | TRINITY_DN5955_c0_g1_i24 | metal ion binding, defense response | Yes | Yes |
|  | TRINITY_DN4194_c0_g1_i9 | DNA-binding transcription factor activity | No | Yes |
|  | TRINITY_DN12696_c0_g1_i1 | phospholipase activity | Yes | Yes |
|  | TRINITY_DN11566_c0_g1_i2 | methyltransferase activity | Yes | Yes |
| Blue | TRINITY_DN25216_c0_g1_i2 | oxidoreductase activity | No | Yes |
|  | TRINITY_DN7687_c0_g1_i6 | transmembrane transport | Yes | Yes |
|  | TRINITY_DN2035_c0_g1_i3 | galactinol-sucrose galactosyltransferase activity | Yes | Yes |
|  | TRINITY_DN62_c0_g3_i1 | protein kinase activity | Yes | Yes |
|  | TRINITY_DN8242_c0_g1_i9 | endo-1,3-beta-D-glucosidase activity | Yes | Yes |
| Black | TRINITY_DN12123_c1_g1_i5 | oxidoreductase activity | Yes | Yes |
|  | TRINITY_DN1681_c0_g1_i3 | acyltransferase activity | Yes | Yes |
|  | TRINITY_DN8000_c0_g1_i1 | amino acid transporter | Yes | Yes |
|  | TRINITY_DN550_c0_g1_i7 | ABC-2 type transporter | Yes | Yes |
|  | TRINITY_DN11525_c0_g1_i1 | Chitinase activity | Yes | Yes |
| Tan | TRINITY_DN4512_c0_g1_i3 | monodehydroascorbate reductase (NADH) activity | Yes | Yes |
|  | TRINITY_DN2829_c0_g1_i10 | transmembrane transporter activity | Yes | Yes |
|  | TRINITY_DN5078_c0_g1_i3 | oxidoreductase activity | Yes | Yes |
|  | TRINITY_DN14647_c0_g1_i1 | transmembrane transporter activity | Yes | Yes |
|  | TRINITY_DN6850_c0_g1_i1 | chitinase activity, defense response to fungus | Yes | Yes |
| Greenyellow | TRINITY_DN7297_c1_g1_i18 | protein kinase activity | Yes | Yes |
|  | TRINITY_DN6750_c0_g1_i12 | Leucine Rich Repeat, protein binding | Yes | Yes |
|  | TRINITY_DN7030_c0_g1_i3 | 3'-5'-exodeoxyribonuclease activity | Yes | Yes |
|  | TRINITY_DN2919_c0_g1_i18 | zinc ion binding, DNA binding | Yes | Yes |
|  | TRINITY_DN11031_c0_g1_i5 | transcription coregulator activity | Yes | Yes |
| Red | TRINITY_DN11120_c0_g1_i3 | Leucine rich repeat N-terminal domain | Yes | Yes |
|  | TRINITY_DN914_c0_g2_i1 | 1-phosphatidylinositol 4-kinase activity | Yes | Yes |
|  | TRINITY_DN820_c0_g3_i5 | pectinesterase activity; cell wall modification | Yes | Yes |
|  | TRINITY_DN14548_c0_g1_i3 | oxidoreductase activity | Yes | Yes |
|  | TRINITY_DN6054_c0_g2_i1 | DNA-binding transcription factor activity | Yes | Yes |

Supplementary Table 4. Summary of the 28 consistently up-regulated genes across different treatments and timepoints in ’14-4’.

| ID | If ortholog to Guardian | If ortholog to MP-29 | Annotation |
| --- | --- | --- | --- |
| TRINITY_DN24283_c0_g1_i2 | Yes | Yes | BAG family molecular chaperone regulator |
| TRINITY_DN2857_c0_g1_i19 | Yes | Yes | cellular response to hypoxia |
| TRINITY_DN10861_c0_g1_i4 | No | No | Ornithine carbamoyltransferase, chloroplastic |
| TRINITY_DN118_c0_g3_i1 | No | Yes | Amino acid permease |
| TRINITY_DN6346_c0_g1_i6 | No | No | 1-aminocyclopropane-1-carboxylate oxidase homolog |
| TRINITY_DN1438_c1_g1_i1 | Yes | Yes | Peroxidase |
| TRINITY_DN6475_c3_g1_i1 | No | No | Glutaredoxin-C7 |
| TRINITY_DN4464_c0_g1_i9 | No | No | Non-specific lipid-transfer protein 1 |
| TRINITY_DN8714_c0_g1_i5 | No | No | Glucan endo-1,3-beta-glucosidase, basic vacuolar isoform |
| TRINITY_DN6346_c0_g1_i22 | No | No | 1-aminocyclopropane-1-carboxylate oxidase homolog |
| TRINITY_DN103_c0_g1_i24 | Yes | Yes | Amino acid permease |
| TRINITY_DN1668_c0_g1_i61 | Yes | Yes | Probable mannitol dehydrogenase |
| TRINITY_DN909_c0_g1_i3 | No | Yes | 2-alkenal reductase (NADP(+)-dependent) |
| TRINITY_DN3711_c0_g1_i17 | No | No | Thaumatin-like protein |
| TRINITY_DN2051_c0_g2_i3 | No | No | Probable aldo-keto reductase |
| TRINITY_DN6421_c0_g1_i14 | No | No | Probable glutathione S-transferase |
| TRINITY_DN35505_c0_g1_i1 | Yes | Yes | Protein FANTASTIC FOUR |
| TRINITY_DN6421_c0_g1_i11 | No | Yes | Probable glutathione S-transferase |
| TRINITY_DN8195_c0_g1_i3 | Yes | Yes | Alpha carbonic anhydrase |
| TRINITY_DN11839_c0_g1_i9 | Yes | Yes | 12-oxophytodienoate reductase |
| TRINITY_DN5241_c0_g1_i1 | Yes | Yes | intracellular protein transport |
| TRINITY_DN6316_c0_g2_i1 | Yes | Yes | Peroxidase 4 |
| TRINITY_DN63884_c0_g1_i1 | Yes | Yes | Aluminum-activated malate transporter |
| TRINITY_DN7335_c0_g1_i1 | Yes | Yes | Thaumatin-like protein |
| TRINITY_DN49331_c0_g1_i3 | Yes | Yes | CEN-like protein |
| TRINITY_DN11650_c0_g1_i1 | Yes | Yes | Probable aspartic proteinase |
| TRINITY_DN8678_c1_g1_i7 | No | No | Kinesin-like protein NACK |
| TRINITY_DN5405_c0_g2_i1 | Yes | Yes | Probable pectate lyase |

Supplementary Table 5. Summary of GO term enrichment of genes which were up- or down-regulated in ‘14-4’.

| Treatment/time | ID | GeneRatio | qvalue | Description |
| --- | --- | --- | --- | --- |
| 72h_up_mellea | GO:0071669 | 27/477 | 4.99E-11 | plant-type cell wall organization or biogenesis |
|  | GO:0009505 | 22/477 | 9.18E-09 | plant-type cell wall |
|  | GO:0042546 | 21/477 | 3.36E-08 | cell wall biogenesis |
|  | GO:0009832 | 18/477 | 7.35E-08 | plant-type cell wall biogenesis |
|  | GO:0009834 | 14/477 | 1.02E-07 | plant-type secondary cell wall biogenesis |
|  | GO:0048046 | 26/477 | 1.02E-07 | apoplast |
|  | GO:0000272 | 23/477 | 3.19E-07 | polysaccharide catabolic process |
|  | GO:0044036 | 23/477 | 4.68E-07 | cell wall macromolecule metabolic process |
|  | GO:0010410 | 15/477 | 1.36E-06 | hemicellulose metabolic process |
|  | GO:0010383 | 17/477 | 1.91E-06 | cell wall polysaccharide metabolic process |
|  | GO:0045488 | 16/477 | 1.91E-06 | pectin metabolic process |
|  | GO:0010393 | 16/477 | 2.42E-06 | galacturonan metabolic process |
|  | GO:0045490 | 11/477 | 3.28E-06 | pectin catabolic process |
|  | GO:0045491 | 11/477 | 3.55E-05 | xylan metabolic process |
|  | GO:0045492 | 8/477 | 3.93E-05 | xylan biosynthetic process |
| 2w_up_mellea | GO:0047501 | 7/675 | 8.20E-05 | (+)-neomenthol dehydrogenase activity |
|  | GO:0009813 | 14/675 | 8.20E-05 | flavonoid biosynthetic process |
|  | GO:0048046 | 27/675 | 8.20E-05 | apoplast |
|  | GO:0009812 | 14/675 | 0.0001636 | flavonoid metabolic process |
|  | GO:0009734 | 19/675 | 0.00031663 | auxin-activated signaling pathway |
|  | GO:0016628 | 13/675 | 0.00035968 | oxidoreductase activity |
|  | GO:0000307 | 10/675 | 0.00050975 | cyclin-dependent protein kinase holoenzyme complex |
|  | GO:0010074 | 12/675 | 0.00050975 | maintenance of meristem identity |
|  | GO:0046029 | 5/675 | 0.00050975 | mannitol dehydrogenase activity |
|  | GO:1905393 | 12/675 | 0.00050975 | plant organ formation |
|  | GO:0090698 | 11/675 | 0.00053461 | post-embryonic plant morphogenesis |
|  | GO:0004601 | 16/675 | 0.00086779 | peroxidase activity |
|  | GO:0048364 | 20/675 | 0.00116005 | root development |
|  | GO:2000280 | 13/675 | 0.00122064 | regulation of root development |
|  | GO:1902554 | 11/675 | 0.00142204 | serine/threonine protein kinase complex |
| 72h_up_tabescens | GO:0042546 | 17/390 | 8.09E-06 | cell wall biogenesis |
|  | GO:0071669 | 18/390 | 1.34E-05 | plant-type cell wall organization or biogenesis |
|  | GO:0009832 | 14/390 | 2.06E-05 | plant-type cell wall biogenesis |
|  | GO:0008422 | 12/390 | 5.29E-05 | beta-glucosidase activity |
|  | GO:0009834 | 10/390 | 0.0001185 | plant-type secondary cell wall biogenesis |
|  | GO:0015926 | 13/390 | 0.0001185 | glucosidase activity |
|  | GO:0048046 | 19/390 | 0.0001185 | apoplast |
|  | GO:0016679 | 10/390 | 0.00015982 | oxidoreductase activity |
|  | GO:0016682 | 8/390 | 0.0004304 | oxidoreductase activity |
|  | GO:0009414 | 18/390 | 0.00053876 | response to water deprivation |
|  | GO:0009820 | 9/390 | 0.00083677 | alkaloid metabolic process |
|  | GO:0009821 | 5/390 | 0.00152941 | alkaloid biosynthetic process |
|  | GO:0009753 | 10/390 | 0.00181928 | response to jasmonic acid |
|  | GO:0102483 | 7/390 | 0.00216076 | scopolin beta-glucosidase activity |
|  | GO:0046274 | 5/390 | 0.00236751 | lignin catabolic process |
| 2w_up_tabescens | GO:0042597 | 56/433 | 8.40E-47 | periplasmic space |
|  | GO:0030288 | 44/433 | 1.27E-45 | outer membrane-bounded periplasmic space |
|  | GO:0009279 | 42/433 | 1.58E-40 | cell outer membrane |
|  | GO:0000270 | 27/433 | 1.41E-24 | peptidoglycan metabolic process |
|  | GO:0006022 | 33/433 | 8.64E-24 | aminoglycan metabolic process |
|  | GO:0005342 | 39/433 | 3.77E-22 | organic acid transmembrane transporter activity |
|  | GO:0046943 | 38/433 | 2.19E-21 | carboxylic acid transmembrane transporter activity |
|  | GO:0030203 | 27/433 | 4.01E-21 | glycosaminoglycan metabolic process |
|  | GO:0008514 | 40/433 | 1.18E-20 | organic anion transmembrane transporter activity |
|  | GO:0043190 | 22/433 | 3.90E-20 | ATP-binding cassette (ABC) transporter complex |
|  | GO:0009401 | 17/433 | 4.50E-20 | phosphoenolpyruvate-dependent sugar phosphotransferase system |
|  | GO:0015293 | 34/433 | 1.28E-19 | symporter activity |
|  | GO:0015144 | 27/433 | 1.38E-19 | carbohydrate transmembrane transporter activity |
|  | GO:0046677 | 24/433 | 7.15E-19 | response to antibiotic |
|  | GO:0006865 | 33/433 | 1.57E-18 | amino acid transport |
